# Molecular docking and ADMET analysis of hydroxamic acids as HDAC2 inhibitors

**DOI:** 10.6026/97320630015380

**Published:** 2019-05-30

**Authors:** Murad Alsawalha, Srinivasa Rao Bolla, Naresh Kandakatla, Venkatesan Srinivasadesikan, Vishnu Priya Veeraraghavan, Krishna Mohan Surapaneni

**Affiliations:** 1 Department of Chemical and Process Engineering Technology, Jubail Industrial College (JIC), P.O. Box 10099, Jubail Industrial City 31961,Kingdom of Saudi Arabia; 2 Department of Anatomy, College of Medicine, Imam Abdulrahman Bin Faisal University, P.O.Box 2114,Dammam 31451, Kingdom of Saudi Arabia; 3 Department of Chemistry, Sathyabama University, Jeppiaar Nagar, Chennai - 600 119, Tamil Nadu, India, 600119; 4 Department of Applied Chemistry, National Chiao Tung University, Hsinchu, 300, Taiwan; 5 5Division of Chemistry,Department of Sciences and Humanities, Vignan's Foundation for Science, Technology and Research University, Vadlamudi, 522 213,Guntur, Andhra Pradesh, India; 6 6Department of Biochemistry, Saveetha Dental College, Saveetha Institute of Medical and Technical Sciences (SIMATS), Saveetha University, 162, P. H. Road, Velappanchavadi, Chennai - 600 077, Tamil Nadu, India; 7 7Department of Medical Biochemistry, College of Applied Medical Sciences in Jubail (CAMSJ), Imam Abdulrahman Bin Faisal University, Jubail Industrial City 35816, Kingdom

**Keywords:** ADMET, histone deacetylase 2, hydroxamic acids, molecular docking

## Abstract

Histone deacetylase (HDAC2) belongs to the hydrolase family and a promising target for cancers. We reported 96 hydroxamic compounds optimized using hydrogen-donors, hydrophobic and electron withdrawing groups followed by molecular docking studies. The optimized compounds show good LibDock score and H-bond interaction in the active site of HDAC2. We selected 20 compounds as the best HDAC2 inhibitors based on the LibDock score, binding energy and hydrogen bonding. ADMET predictions on these compounds show good absorption, BBB penetration and no liver toxicity. We subsequently report four compounds selected as best HDAC2 inhibitors based on the LibDock, binding energy, H-bonding and ADMET properties.

## Background

Chromatin structure of histone has two forms such as Histone acetylases (HATs) and Histone deacetylases (HDACs). The acetylation status of histone operated by histone acetylases and histone deacetylases, which are in equilibrium 1.The main function of HDAC is deacetylation of e-amino groups of lysine located near the amino terminal of core histone proteins and restore positive charge on lysine residue, which results in tightening of nucleosome structure and gene silencing 2. HDACs not only deacetylate the histone proteins, but also deacetylate non-histone proteins, such as p53 and GATA-1 3. Histone deacetylase protein belongs to the hydrolase family and classified into two classes on the basis of sequence similarity, class I has four isomers of HDAC1- 3, and HDAC8 and are related to yeast Rpd3 gene, class II has six isomers of HDAC4-7 and HDAC9-10 and are related to Hda1 and class I and II operated by zinc dependent mechanism 4. Histone deacetylases (HDACs) control the gene expression and cellular signalling and histone deacetylases 2 (HDAC2) is over expressed in solid tumors including colon cancer, lung cancer, cervical carcinoma, breast cancer, and kidney/cervix cancer and also in Alzheimer's disease 5-7. Several natural and synthetic derivatives have been identified to be able to inhibit the activity of the HDACs.

HDAC inhibitors (HDACi) arrest cell growth and leads to differentiation and apoptosis in tumor cells. HDACi can be divided into several structural classes including hydroxamic acids, cyclic peptides, aliphatic acids and benzamides etc. 8-9. Naturally identified Hydroxamic acid HDAC inhibitor was Trichostatin A (TSA) and SAHA (Suberoylanilide hydroxamic acid or Vorinostat (Zolinza)) is structurally similar to TSA was first HDAC inhibitor approved for the treatment of refractory cutaneous T-cell lymphoma by Food and Drug Administration (FDA) in October 2006 10-11. The compounds with radio sensitizing properties were found to be effective in the clinical application as they are cell specific 12. Research on the SAHA as HDAC inhibitor for the treatment of hematologic and solid tumors is found to be efficient 13. Some studies found that HDAC inhibitors can be used for targeting the radio resistant cancers 14. Trail (Apo2L, TNFSF10) as a mediator tigers the tumor cell death in acute myeloid leukemia 15. Finding the specific HDACi for the individual HDAC is an important goal since HDACs are found to maintain different biological activities. Drug design is one of the emerging and important fields for drug discovery. The studies help in developing novel structures and potent drug molecules used for the drug therapies 16. Studies on Uveal melanoma concluded that HDAC inhibitors provoked morphological differentiation which hindered the growth of tumor 17. SAHA is a low toxic drug that was docked to get 12 different versions by drug modification and was screened. These were evaluated and were found to exhibit more potency and better affinity than the SAHA 18. In order to develop best class of drugs, the innovative approach for drug designing, is opted by the researchers in recent times 19. Optimization of HDAC2 inhibitors of hydroxamic acid was reported previously 20- 21 and in this study we optimized hydroxamic acid group. Based on our previous QSAR and pharmacophore studies, molecular docking, binding energy and ADMET studies were carried out.

## Methodology

### Data set:

**Figure 1:**
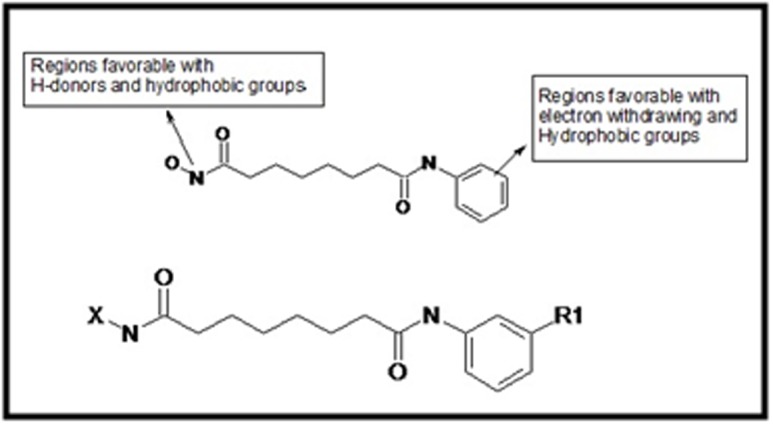
Structural requirement for designing potent hydroxamic acids inhibitors

Hydroxamic acids were optimized to improve the inhibitory activity towards HDAC2 protein. SAHA was chosen as reference structure to design new set of compounds. Total 96 compounds were designed based on the 3D-QSAR model on hydroxamic acid (Figure 1) 22. Hydroxamic acid derivatives were optimized with H-bond donors (OH, CH3, CH=CH2, Ph), hydrophobic (hydrocarbons - Pyrrole, Furan, Thiophene, Imidazole, Oxazole, Isooxzole, Benzene, Ph-NO2, Ph-COCH3, Ph-CCl3, Aniline, Indole, Pyridine and Pyrimidine) and electron withdrawing groups (NO2, NCH3, SO3CH3, COCl, COOH, COCH3, COH, Br, Cl and F) were listed in Table 1. Molecular docking analysis performed on these molecules to investigate for better HDAC2 inhibitors. All ligands were sketched using ISIS draw and given as input file in prepare ligand module in Discovery studio (DS). This generated 3Dstructures, tatuomers, and isomers and filtered the ligands by Lipinski rule of five. After applying the force fields on ligands the structures were minimized for lowest energy.

### Protein preparation and docking:

The crystal structure of HDAC2 (PDB ID: 3MAX) was downloaded from protein database (http://www.rcsb.org/pdb). The protein preparation was carried out in DS by removing water molecules and co-crystallized ligand further applying force filed parameter CHARMm to protein. The receptor binding sites were searched using flood filling algorithm. Docking calculations carried out using LibDock program implemented in discovery studio 23. The 15 - site sphere was selected using coordinates in predefined binding site for docking studies. The 500 binding site features, so call 'HotSpots' in binding site spheres were determined using a grid placed into the binding site with polar and apolar probes. The conformations of ligands poses were generated using FAST method and then placed into the binding site sphere. The docking poses were pruned and optimized. Final best optimized compounds were selected based on the LibDock score and H-bonds and the results were compared with the SAHA compound.

In 3D molecular docking studies, the candidate compound docked into the target protein and provides a variety of structural information such as hydrogen bonding interaction, electrostatic interaction, and molecular surface complementary and so on. The binding energy of complex calculated using Eq. 1, which gives the better understanding of binding affinity of the docked complex.

ΔE = E complex - (E enzyme + E ligand) (Eq. 1)

Best resulted compounds from molecular docking studies were considered for binding energy calculations. The energies of each inhibitor and HDAC2 were calculated by semi-empirical method PM6 24. The energy association of each ligand (ΔE) was estimated by three types of calculations such as (i) single point energy calculations of active site residues of protein and inhibitor complex (E complex) (ii) energy calculation of chosen active site residue of protein (E enzyme)and (iii) single point energy calculation on ligand (E ligand). The PM6 method used in this study because of the size of the complex and also the binding/interaction energies reported using PMx method shows good results 25-30. The quantum chemical calculations were performed using GAUSSIAN 09 31.

### ADMET:

ADMET (Absorption, Distribution, Metabolism, and Excretion): In drug discovery many potential drugs failed in clinical trials or late drug discovery process, due to poor drug like properties and adverse side effects. In the current investigation, all the optimized hydroxamic acid compounds were subjected to ADMET studies to make sure toxicity risks and drug-relevant properties of molecules which are key factors, to determine drug-likeness of lead molecules. ADMET studies were conducted on selected lead compounds using Discovery Studio (Accelrys, San Diego, CA, USA). This module uses six mathematical models, to quantitatively predict properties by a set of rules/keys that specify threshold ADMET characteristics for the chemical structure of the molecules based on the available drug information.

## Results and Discussion:

### Molecular docking:

**Figure 2:**
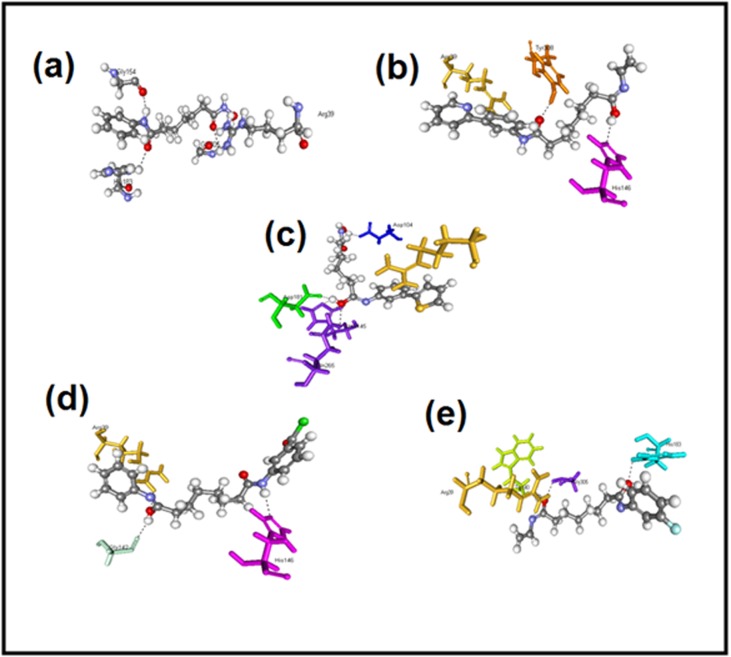
Docking poses of top four compounds (a) SAHA (b) (3-(8-oxo-8-(phenylamino)octanamido)benzoyl chloride (H34); (c) N1-(3- (pyridin-2-yl)phenyl)-N8-vinyloctanediamide (H81); (d) N1-hydroxy-N8-(3-(thiophen-2-yl)phenyl)octanediamide (H43); (e) N1-(3- fluorophenyl)-N8-vinyloctanediamide) (H30).

Molecular docking studies were carried out on 96 designed hydroxamic acids from 3D-QSAR studies. The HDAC2 protein has 3 chains (Chain A, B and C), chain A is selected for docking studies 20, 32. LibDock score, binding energy and H bonding considers for selection of best HDAC2 inhibitors. SAHA is chosen as reference compound for comparing the docking score of compounds. SAHA has the LibDock score of 126.37 dock score and 4 hydrogen bond interactions with ARG39(2), HIS183, GLY305, GLY154 amino acids and pi-pi interaction with PHE155. About 62 compounds among 96 were shown good docking score than SAHA, top listed 20 compounds with LibDock score and H-bonds were shown in Table 2. Based on molecular docking and H-bond interaction four compounds are selected as best inhibitor of HDAC2 protein. H34 (3-(8-oxo-8-(phenylamino) octanamido) benzoyl chloride) has the LibDock score of 153.22 and three Hbonds with ARG39, HIS146, GLY142 and pi-pi bond with ARG39 shown in Figure 2 (b), It shows oxygen of N-hydroxyl group forms H-bonds with HIS146, oxygen of formamide forms H-bonds with GLY142 and ARG39. LibDock score 145.94 for H81 (N1-(3-(pyridin- 2-yl)phenyl)-N8-vinyloctanediamide) with three H-bonds with ARG39, TYR308, HIS146 and pi-pi bond with ARG39 shown in Figure 2(c), it shows oxygen of N-hydroxyl group forms H-bonds with HIS146, oxygen of formamide forms H-bond with TYR308 and ARG39. H43 (N1-hydroxy-N8-(3-(thiophen-2-yl) phenyl) octanediamide) has LibDock score of 145.85 and five H-bonds with ARG39, GLN265 (2), HIS145, ASP181, ASP104 and pi-pi bond with ARG39 shown in Figure 2(d), It shows oxygen of N-hydroxyl group forms H-bonds with ASP104, oxygen of formamide forms H-bonds with ASP181, HIS145 and GLN265, thiophene of sulphar forms bonds with ARG39. H30 (N1-(3-fluorophenyl)-N8-vinyl octanediamide) has LibDock score of 143.00 with four H-bonds ARG39 (2), HIS183, GLY305, TRP140 and pi-pi bond with PHE155 shown in Figure 2 (e), It shows oxygen of N-hydroxyl group forms H-bonds with ARG39, GLY305 and TRP40 and oxygen of formamide forms H-bond with HIS183.The result shows that Nhydroxyl group, which is an important group and forms interactions with the HDAC2.

### Binding Energy calculation:

Binding energy calculations were performed on the best 20 Compounds which have good docking score and H-bond interaction and results are listed in Table 2. In order to compare the obtained Binding energy (ΔE), calculations also performed on active HDAC2 inhibitors (SAHA). Table 2 shows the binding energy calculation (PM6) of HDAC2-inhibitor complexes. The binding energy of active HDAC2 inhibitors SAHA is -33.25 kcal/mol, and the binding energy of selected four compounds as (3-(8-oxo-8-(phenylamino) octanamido)benzoyl chloride (H34) (- 39.53 kcal/mol), N1-(3-(pyridin-2-yl)phenyl)-N8- vinyloctanediamide (H81) (-56.08 kcal/mol), N1-hydroxy-N8-(3- (thiophen-2-yl)phenyl) octanediamide (H43) (-43.21 kcal/mol), N1- (3-fluorophenyl)-N8-vinyloctanediamide) (-35.76 kcal/mol), it shows these compounds have smaller binding energy than active HDAC2 inhibitors and were suggesting an inhibitors of HDAC2.The compounds, which are having hydrogen bond interactions with ARG, HIS, TYR active residues shows smaller binding energies. This implies that the active site residues ARG, HIS, TYR are become more favourable to the binding of HDAC2 inhibitors.

### ADMET:

**Figure 3:**
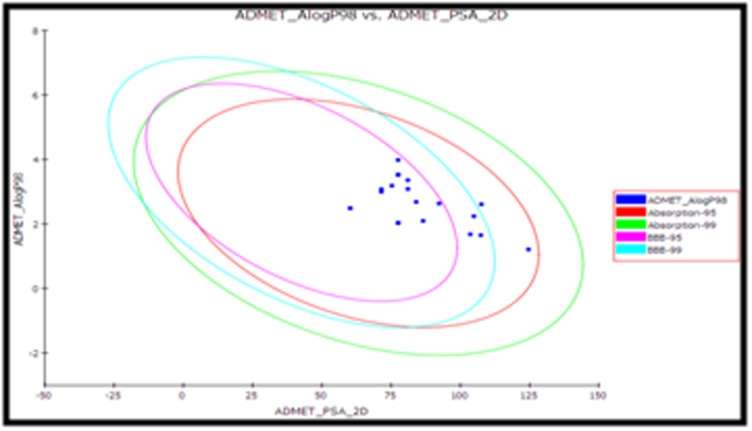
Plot of Polar Surface Area (PSA) vs. LogP for a standard and test set showing the 95% and 99% confidence limit ellipses corresponding to the Blood Brain Barrier and Intestinal Absorption models.

ADMET predictions were carried out to evaluate drug likeness of top 20 selected compounds and the properties were reported in Table 3 together with biplot Figure 3. The pharmacokinetic profiles of selected compounds were predicted by means of six precalculated ADMET model provided by Discovery studio. Figure 3 bi plot shows two analogous 95% and 99% confidence ellipse corresponding to HIA and BBB models. PSA have inverse relationship with human intestinal absorption and thus cell wall permeability. The log P used to estimate the lipophilicity, thus the information of H-bonding characteristics as obtained by calculating PSA could be taken into consideration along with logP calculation. The model with descriptors AlogP98 and PSA 2D with a bi-plot comprising 95% and 99% confidence ellipses was considered for the accurate prediction for the cell permeability of compounds. Selected 20 compounds had a good adsorption prediction for metabolism. In toxicity evaluation except H32 all compounds displayed CYP2D6 inhibiting and hepatotoxicity, suggesting that these compounds have no toxicity in the liver. Blood brain barrier (BBB) penetration showed that 10 compounds have good penetration; 8 compounds have low penetration and 2 compounds have undefined penetration;10 compounds may suitable for central nerve system therapy. Four compounds (3-(8-oxo-8- (phenylamino)octanamido)benzoyl chloride (H34); N1-(3-(pyridin- 2-yl)phenyl)-N8-vinyloctanediamide (H81);N1-hydroxy-N8-(3- (thiophen-2-yl)phenyl)octanediamide (H43); N1-(3-fluorophenyl)- N8-vinyloctanediamide) (H30) were selected as potential compounds based on the LibDock, binding energy, H bonding and ADMET properties.

## Conclusion

It is of interest to identify better inhibitors for HDAC2. Here, we report the binding of 4 HDAC2 inhibitors with optimal LibDock score, binding energy and hydrogen-bonds. It is further noted by ADMET analysis that these compounds have good absorption, less toxic in the human liver and BBB penetration and may therefore suggest as HDAC2 inhibitors.

## Figures and Tables

**Table 1:** Different substitutions used in hydroxamic acid derivatives

		R1			R1
Compd No	X	(EW, hydrocarbon)	Compd No	X	(EW, hydrocarbon)
H1	N-OH	NO2	H49	N-OH	Ph-COCH3
H2	N-OH	NCH3	H50	N-OH	Ph-CCl3
H3	N-OH	SO3CH3	H51	N-OH	Aniline
H4	N-OH	COCl	H52	N-OH	Indole
H5	N-OH	COOH	H53	N-OH	Pyridine
H6	N-OH	COCH3	H54	N-OH	pyrimidine
H7	N-OH	COH	H55	N-CH3	pyrrole
H8	N-OH	Br	H56	N-CH3	furan
H9	N-OH	Cl	H57	N-CH3	thiophene
H10	N-OH	F	H58	N-CH3	Imidazole
H11	N-CH3	NO2	H59	N-CH3	Oxazole
H12	N-CH3	NCH3	H60	N-CH3	Isooxzole
H13	N-CH3	SO3CH3	H61	N-CH3	Benzene
H14	N-CH3	COCl	H62	N-CH3	Ph-NO2
H15	N-CH3	COOR	H63	N-CH3	Ph-COCH3
H16	N-CH3	COR	H64	N-CH3	Ph-CCl3
H17	N-CH3	COH	H65	N-CH3	Aniline
H18	N-CH3	Br	H66	N-CH3	Indole
H19	N-CH3	Cl	H67	N-CH3	Pyridine
H20	N-CH3	F	H68	N-CH3	pyrimidine
H21	N-CH=CH2	NO2	H69	N-CH=CH2	pyrrole
H22	N-CH=CH2	NCH3	H70	N-CH=CH2	furan
H23	N-CH=CH2	SO3CH3	H71	N-CH=CH2	thiophene
H24	N-CH=CH2	COCl	H72	N-CH=CH2	Imidazole
H25	N-CH=CH2	COOR	H73	N-CH=CH2	Oxazole
H26	N-CH=CH2	COR	H74	N-CH=CH2	Isooxzole
H27	N-CH=CH2	COH	H75	N-CH=CH2	Benzene
H28	N-CH=CH2	Br	H76	N-CH=CH2	Ph-NO2
H29	N-CH=CH2	Cl	H77	N-CH=CH2	Ph-COCH3
H30	N-CH=CH2	F	H78	N-CH=CH2	Ph-CCl3
H31	N-Ph	NO2	H79	N-CH=CH2	Aniline
H32	N-Ph	NCH3	H80	N-CH=CH2	Indole
H33	N-Ph	SO3CH3	H81	N-CH=CH2	Pyridine
H34	N-Ph	COCl	H82	N-CH=CH2	pyrimidine
H35	N-Ph	COOR	H83	N-Ph	pyrrole
H36	N-Ph	COR	H84	N-Ph	furan
H37	N-Ph	COH	H85	N-Ph	thiophene
H38	N-Ph	Br	H86	N-Ph	Imidazole
H39	N-Ph	Cl	H87	N-Ph	Oxazole
H40	N-Ph	F	H88	N-Ph	Isooxzole
H41	N-OH	Pyrrole	H89	N-Ph	Benzene
H42	N-OH	Furan	H90	N-Ph	Ph-NO2
H43	N-OH	thiophene	H91	N-Ph	Ph-COCH3
H44	N-OH	Imidazole	H92	N-Ph	Ph-CCl3
H45	N-OH	Oxazole	H93	N-Ph	Aniline
H46	N-OH	Isooxzole	H94	N-Ph	Indole
H47	N-OH	Benzene	H95	N-Ph	Pyridine
H48	N-OH	Ph-NO2	H96	N-Ph	pyrimidine

**Table 2:** LibDock, Binding energy and H-bond interactions of hydroxamic acids

Comp	LibDock Score	Binding Energy (Kcal/mol)	H-Bonds	H-Bond Monitor	H-Bond distance
SAHA	126.37	-33.25	ARG39(2), HIS183, GLY305, GLY154	P:ARG39:HH21 -L:O19	2.40, 2.14, 2.18, 2.46, 1.92
				P:ARG39:HH22 - L:O18	
				P:HIS183:HD1 - L:O10	
				P:GLY305:HN - L:O18	
				L:H25 - P:GLY154:O	
H32	162.45	-18.78	ARG39, GLY142	P: ARG39: HH22 - L:O9	2.40, 2.18
				L:H31 - P:GLY142:O	
H51	153.359	-30.16	HIS183, ASP181(2), TYR209, LEU276	P:HIS183:HD1 - L:O10	2.24, 2.12, 2.18, 1.78, 2.49
				L:H33 - P:ASP181:OD1	
				L:H33 - P:ASP181:OD2	
				L:H45 - P:TYR209:OH	
				L:H45 - P:LEU276:O	
H34	153.221	-39.53	ARG39, HIS146, GLY142	P: ARG39:HH22 - L:O10	2.34, 2.20, 2.00
				L:H28 - P:HIS146:NE2	
				L:H30 - P:GLY142:O	
H36	152.171	-36.9	TYR29(2), HIS183, TYR29	P:TYR29:HH - L:O9	2.31, 1.72, 2.04
				P:HIS183:HD1 - L:O8	
				L:H30 - P:TYR29:OH	
H53	147.078	-39.72	ARG39, GLY305, HIS146,	P:ARG39:HH21 - L:O18	1.75, 2.12, 2.07, 2.39, 1.88
			GLY142(2)	P:GLY305:HN - L:O19	
				L:H30 - P:HIS146:NE2	
				L:H43 - P:GLY142:O	
				L: H44 - P:GLY142:O	
H81	145.945	-56.08	ARG39, TYR308, HIS146	P:ARG39:HH21 - L:N5	1.81, 2.34, 2.03
				P:TYR308:HH - L:O9	
				L:H29 - P:HIS146:NE2	
H43	145.859	-45.1	ARG39, GLN265(2), HIS145, ASP181, ASP104	P:ARG39:HH21 - L:S24	2.44, 2.46, 2.14, 2.40, 2.07, 1.89
				P:GLN265:HE21 - L:O10	
				P:GLN265:HE22 - L:O10	
				L:H31 - P:HIS145:NE2	
				L:H31 - P:ASP181:OD1	
				L:H43 - P:ASP104:OD2	
H72	145.32	-10.3	HIS183, TYR308	L:H26 - P:HIS183:NE2	2.15, 2.02
				L:H27 - P:TYR308:OH	
H67	144.667	-8.4	HIS183, GLY154	P: HIS183:HD1 - L: O9	2.37, 1.91
				L: H26 - P: GLY154:O	
H74	144.355	-5.02	ARG39, GLY305, GLY154, GLY142	P: ARG39:HH21 - L: N14	2.29, 2.49, 1.92, 1.73
				P: GLY305: HN - L:O13	
				L:H27 - P:GLY154:O	
				L: H30 - P: GLY142:O	
H54	144.304	-33.57	ARG39, GLN265, GLY306, ASP181	P:ARG39:HH21 - L:N25	2.36, 2.35, 2.37, 1.90, 1.93
				P:GLN265:HE22 - L:O10	
				P:GLY306:HN - L:O10	
				L:H32 - P:ASP181:OD1	
H27	144.161	-40.78	ARG39(3), HIS183(2), GLY305, GLY142	P:ARG39:HH21 -L:N7	2.27, 2.40, 2.45, 2.40, 1.95, 2.44, 1.74
				P:ARG39:HH22 - L:O9	
				P:ARG39:HH22 - L:O6	
				P:HIS183:HD1 - L:O8	
				P:HIS183:HD1 - L:O5	
				P:GLY305:HN - L:O6	
				L:H24 - P:GLY142:O	
H46	143.584	-26.2	ARG39, HIS145, GLY143, ASP104	P: ARG39:HH21 - L: N24	2.36, 2.46, 1.80, 2.21
				P: HIS145: HN - L: O10	
				L: H31 - P:GLY143:O	
				L:H43 - P:ASP104:OD2	
H37	143.211	-2.03	ARG39 (2)	P:ARG39:HH21 - L:O6	2.31, 2.12
				P:ARG39:HH22 - L:O6	
H30	143.005	-35.76	ARG39(2), HIS183, GLY305, TRP140	P:ARG39:HH21 - L:N8	2.16, 2.45, 2.13, 2.18, 1.85
				P:ARG39:HH22 - L:O7	
				P:HIS183:HD1 - L:O6	
				P:GLY305:HN - L:O7	
				L:H24 - P:TRP140:O	
H26	142.892	-26.98	ARG39, GLY305, TYR308, GLY142	P:ARG39:HH21 - L:N11	2.20, 2.40, 1.97, 1.73
				P:GLY305:HN - L:O9	
				L:H24 - P:TYR308:OH	
				L:H26 - P:GLY142:O	
H47	142.128	-36.92	GLN265, GLY306, ASP181, ASP104	P:GLN265:HE22 - L:O10	2.45, 2.38, 2.05, 1.84
				P:GLY306:HN - L:O10	
				L:H32 - P:ASP181:OD1	
				L:H44 - P:ASP104:OD2	
H3	141.334	-20.6	ARG39, TYR308, HIS183	P:ARG39:HH21 - L:O22	1.75, 1.92, 2.09
				P:TYR308:HH - L:O10	
				L:H42 - P:HIS183:NE2	
H69	141.308	-8.06	TYR308, ALA141	P:TYR308:HH - L:O12	2.17, 1.84
				L:H26 - P:ALA141:O	
H44	141.29	-57.1	ARG39, TYR308, GLY142, ALA141, HIS183	P:ARG39:HH21 - L:O18	2.18, 1.93, 2.21, 1.82, 2.08
				L:H29 - P:TYR308:OH	
				L:H42 - P:GLY142:O	
				L:H43 - P:ALA141:O	
				L:H46 - P:HIS183:NE2	

**Table 3:** ADMET prediction of top 20 optimized compounds

Compound	aAbsorption	bBBB Level	cCYP2D6	dHepatotoxicity
Compound 3	0	4	1	0.509
Compound 26	0	3	1	0.536
Compound 27	0	3	1	0.509
Compound 30	0	2	1	0.476
Compound 32	0	2	0	0.582
Compound 34	0	2	1	0.701
Compound 36	0	2	1	0.688
Compound 37	0	2	1	0.642
Compound 43	0	2	1	0.602
Compound 44	0	3	1	0.49
Compound 46	0	3	1	0.437
Compound 47	0	2	1	0.662
Compound 51	0	4	1	0.682
Compound 53	0	3	1	0.682
Compound 54	0	3	1	0.655
Compound 67	0	2	1	0.509
Compound 69	0	2	1	0.596
Compound 72	0	3	1	0.503
Compound 74	0	3	1	0.443
Compound 810	0	2	1	0.649
